# Supplementary material for: Splice-Junction-Based Mapping of Alternative Isoforms in the Human Proteome
Source: Cell Rep. Author manuscript; Available in PMC 2020 Jan 15. (PMC6961840; doi:10.1016/j.celrep.2019.11.026)
Supplement: 2 [file NIHMS1546469-supplement-2.zip › DF1/PXD010154/Thyroid/Thyroid_17_TG_CERPQLWQTIQTQGHFQLQLPPGK.pdf]

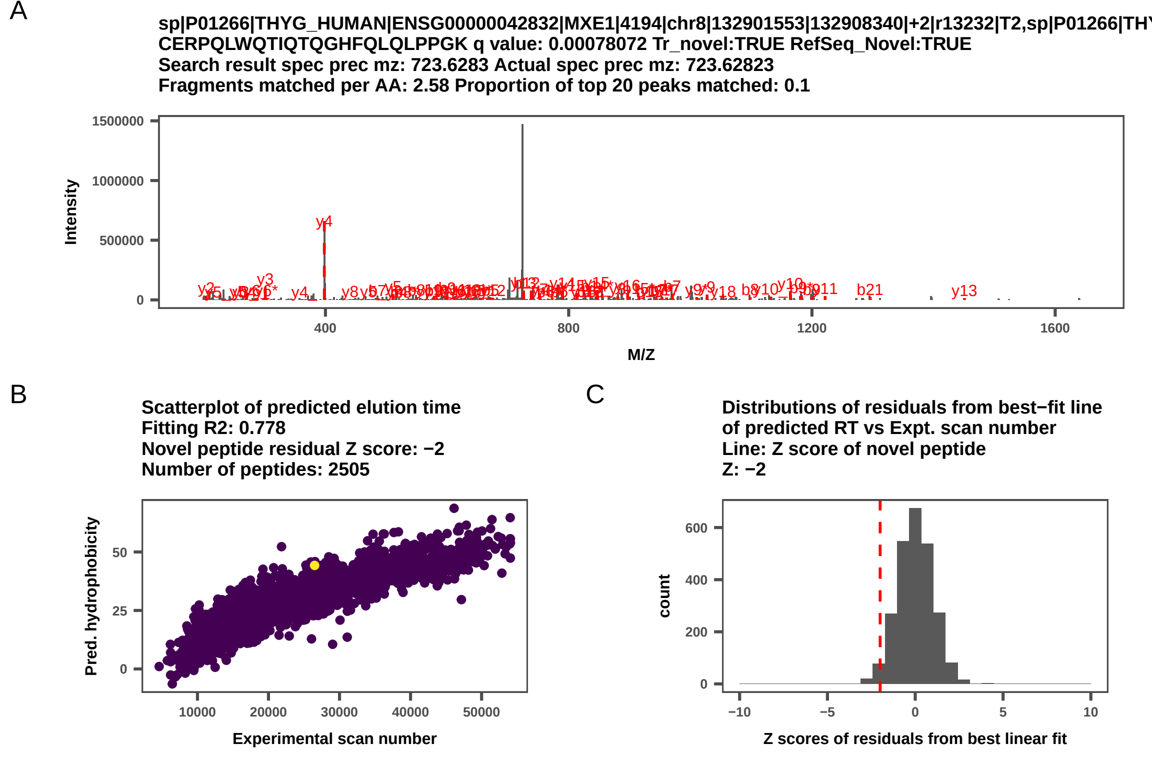
A
sp|P01266|THYG_HUMAN|ENSG00000042832|MXE1|4194|chr8|132901553|132908340|+2|r13232|T2,sp|P01266|THY
CERPQLWQTIQTQGHFQLQLPPGK q value: 0.00078072 Tr_novel:TRUE RefSeq_Novel:TRUE
Search result spec prec mz: 723.6283 Actual spec prec mz: 723.62823
Fragments matched per AA: 2.58 Proportion of top 20 peaks matched: 0.1
Intensity
1500000
1000000
500000
0
y4
y3
y2
b21
y13
400
800
1200
1600
M/Z
B
Scatterplot of predicted elution time
Fitting R2: 0.778
Novel peptide residual Z score: −2
Number of peptides: 2505
Pred. hydrophobicity
50
25
0
10000
20000
30000
40000
50000
Experimental scan number
C
Distributions of residuals from best−fit line
of predicted RT vs Expt. scan number
Line: Z score of novel peptide
Z: −2
count
600
400
200
0
−10
−5
0
5
10
Z scores of residuals from best linear fit
